# Primary and tertiary health professionals’ views on the health-care of patients with co-morbid diabetes and chronic kidney disease – a qualitative study

**DOI:** 10.1186/s12882-016-0262-2

**Published:** 2016-05-18

**Authors:** Clement Lo, Dragan Ilic, Helena Teede, Greg Fulcher, Martin Gallagher, Peter G Kerr, Kerry Murphy, Kevan Polkinghorne, Grant Russell, Timothy Usherwood, Rowan Walker, Sophia Zoungas

**Affiliations:** Monash Centre for Health Research and Implementation, School of Public Health and Preventive Medicine, Monash University, Clayton, Victoria Australia; Diabetes and Vascular Medicine Unit, Monash Health, Clayton, Victoria Australia; Department of Epidemiology & Preventive Medicine, School of Public Health and Preventive Medicine, Monash University, Prahran, Victoria Australia; Department of Diabetes and Endocrinology, Royal North Shore Hospital, Sydney, NSW Australia; The George Institute for Global Health, Sydney, NSW Australia; Department of Nephrology, Concord Hospital, Sydney, NSW Australia; Department of Nephrology, Monash Health, Clayton, Victoria Australia; School of Primary Health Care, Monash University, Clayton, Victoria Australia; Department of General Practice, Westmead Clinical School, University of Sydney, Sydney, NSW Australia; Department of Renal Medicine, Alfred Health, Prahran, Victoria Australia

**Keywords:** Qualitative, Focus groups, Multi-morbidity, Diabetes, Chronic kidney disease, Health-care, Health-care delivery, Primary care, Tertiary care

## Abstract

**Background:**

Health-care for co-morbid diabetes and chronic kidney disease (CKD) is often sub-optimal. To improve health-care, we explored the perspectives of general practitioners (GPs) and tertiary health-care professionals concerning key factors influencing health-care of diabetes and CKD.

**Methods:**

A total of 65 health professionals were purposively sampled from Australia’s 2 largest cities to participate in focus groups and semi-structured interviews. Four focus groups were conducted with GPs who referred to 4 tertiary health services in Australia’s 2 largest cities, with 6 focus groups conducted with tertiary health-care professionals from the 4 tertiary health services. An additional 8 semi-structured interviews were performed with specialist physicians who were heads of diabetes and renal units. All discussions were facilitated by the same researcher, with discussions digitally recorded and transcribed verbatim. All qualitative data was thematically analysed independently by 2 researchers.

**Results:**

Both GPs and tertiary health-care professionals emphasised the importance of primary care and that optimal health-care was an inter-play between patient self-management and primary health-care, with specialist tertiary health-care support. Patient self-management, access to specialty care, coordination of care and a preventive approach were identified as key factors that influence healthcare and require improvement. Both groups suggested that an integrated specialist diabetes-kidney service could improve care. Unit heads emphasised the importance of quality improvement activities.

**Conclusions:**

GPs and tertiary health-care professionals emphasised the importance of patient self-management and primary care involvement in the health-care of diabetes and CKD. Supporting GPs with an accessible, multidisciplinary diabetes-renal health service underpinned by strong communication pathways, a preventive approach and quality improvement activities, may improve health-care and patient outcomes in co-morbid diabetes and CKD.

**Electronic supplementary material:**

The online version of this article (doi:10.1186/s12882-016-0262-2) contains supplementary material, which is available to authorized users.

## Background

The 2015 Global Burden of Disease Study report indicated that people are living longer but with more multi-morbidity and increased disability [1, 2] predominantly driven by chronic non-communicable diseases, including diabetes. Diabetes is a major cause of chronic disease with 8.3 % of adults, or 382 million people, estimated in 2013 to be affected worldwide [3]. It is also the leading cause of chronic kidney disease (CKD), accounting for up to 50 % of people who develop end-stage kidney disease, and can co-exist with non-diabetic CKD [4]. Multi-morbidity due to diabetes and CKD substantively increases disability and mortality, especially due to cardiovascular disease [5]. The annual estimated Medicare fee for service costs for diabetes and CKD for those aged greater than 65 years old has been estimated at 13.6 billion USD in America in 2013 [6].

There has been emerging evidence of suboptimal management of co-morbid diabetes and CKD with common problems including the failure to identify renal disease resulting in inappropriate usage and dosage of medications, adverse medication effects [7–10], and delayed referral to specialists [11]. Other problems include the failure to meet clinical performance parameters such as blood pressure [8] and glycaemic targets [12], the lack of appropriate use of angiotensin blockade agents and statins [8], and poor screening of cardiovascular risk factors [12] and diabetic complications [8, 12].

This may reflect the outcomes of health systems poorly equipped to deal with and manage multi-morbidity in general [2] and co-morbidity due to diabetes and CKD specifically. People with multi-morbidity require a broader approach than the individual chronic disease focus which configures the delivery of most health-care systems and dominates research globally [2, 13]. Additionally, while people with co-morbid diabetes and CKD emphasise the importance of self-management, disease related adverse experiences due to co-morbidity, especially in later stages of CKD (such as tiredness, feeling unwell and increased disability) and the related psychological sequelae can hinder self-management [14], and should be recognised and addressed in health service delivery.

Given multi-morbidity is likely to become an increasing challenge as health-systems serve an aging population with more chronic diseases [1, 2], research targeting health-care improvement for multi-morbidity, such as co-morbid diabetes and CKD, should be a priority. While previous research has mainly taken a deductive approach testing a model of health delivery [10, 15, 16] there has been a paucity of research adopting an inductive and formative approach exploring the ideas and priorities of people involved in delivering or receiving health-care for co-morbid diabetes and chronic kidney disease.

In Australia, most specialist ambulatory health-care is provided free at government-funded public hospitals or subsidised by the government through a universal health care scheme called Medicare. In this setting, general practitioners (GPs) are the gateway to tertiary health-care or specialist health professionals and allied health professionals with GP referrals required to access specialist tertiary health-care and allied health-care. As such the majority of the health-care of patients with co-morbid diabetes and CKD is provided by primary (GPs) and tertiary health-care professionals.

In this qualitative study, we explored how the health-care of patients with co-morbid diabetes and CKD can be improved by examining the perceptions of primary and tertiary health-care professionals concerning the key factors influencing optimal health-care for this patient population.

## Methods

This qualitative study was a research collaboration between 4 large tertiary health services across 2 of Australia’s largest cities, 2 research institutes (Monash Centre for Health Research and Implementation and The George Institute for Global Health) and 2 national consumer stakeholder groups (Diabetes Australia and Kidney Health Australia). The study was underpinned by a pragmatic worldview [17], whereby the emphasis is using available effective methods to explore and find a solution to a research problem, rather than fidelity to a single system of philosophy and reality [18].

We utilised focus groups of GPs and tertiary health-care professionals (excluding specialist unit heads) to explore a wide range of issues and perspectives, which is less likely to occur in a semi-structured interview dynamic [19, 20]. Separate focus groups were held for tertiary health-care professionals working at each tertiary health service (Alfred Health, Concord Hospital, Monash Health and the Royal North Shore Hospital) and for GPs working in the health region of each tertiary health service, ensuring that GPs and tertiary health professionals did not influence each other’s views. Additionally, specialist physicians who were diabetes and renal unit heads from each tertiary health service participated in semi-structured interviews separate from the focus groups, ensuring that tertiary health professionals could share their perspectives in an unhindered manner and allowing triangulation or cross-referencing of results with findings of the focus groups. The study was approved by all local hospital and university Human Research Ethics Committees (Monash Health Human Research Ethics Committee, Alfred Health Research Ethics Committee, Monash University Human Research Ethics Committee, Northern Sydney Local Health District Human Research Ethics Committee, Sydney Local Health District Human Research Ethics Committee and the University of Sydney Human Research Ethics Committee).

### Participant selection and setting

GPs were recruited from the health regions associated with the 4 tertiary health services. We sought to recruit a representative sample of GPs by faxing a letter of invitation to all GP clinics in the 4 regions (598 GPs) followed up by phone calls. Respondents to the fax and/or phone call attended focus groups in each region.

Tertiary health professionals from 4 tertiary health-care services in 2 of Australia’s most populous cities (Alfred and Monash Health in Melbourne, and Royal North Shore and Concord Hospitals in Sydney) were purposively sampled based on each diabetes and renal unit’s head physician’s knowledge and network of information rich cases. Fifty-one tertiary health professionals were approached by an independent research assistant or a researcher (CL) and 35 were able to participate. Maximal variation sampling ensured representation of endocrine, renal and allied health professionals [20]. Physicians who were the diabetes and renal unit heads in each hospital were also recruited for semi-structured interviews. All who were contacted (8 in total), agreed to participate.

Participants were assured of the de-identification and confidentiality of all data and participated voluntarily and gave written consent for participation in and audio-recording of focus groups. Participants who were GPs were remunerated as per standard health service approaches in Australia however specialists were not remunerated. Focus groups were conducted in a meeting room at the main hospital of each tertiary health-service. Semi-structured interviews were conducted in the office of the diabetes or renal unit’s head physician.

### Data collection

Open ended discussion questions for both focus groups and semi-structured interviews (Additional file 1: Table S1) were developed after a literature review (of health-care of co-morbid diabetes and chronic kidney disease) in consultation with the research team and piloted as a semi-structured interview with 4 Endocrinologists from Monash Health. An iterative approach was used with additional questions added according to themes raised in preceding focus groups and semi-structured interviews. Focus group and semi-structured interviews were conducted and digitally audio recorded by the same researcher (CL, a male Endocrinologist, with training and experience in conducting focus groups and semi-structured interviews) from May 2013 to February 2014. Focus groups and semi-structured interviews were conducted until a point of data saturation was reached, with no new ideas emerging. This occurred after conducting 10 focus groups and 8 semi-structured interviews with a total of 65 participants. De-identified audiotaped discussions were transcribed verbatim by an independent transcribing service.

### Data analysis

Data analysis was an iterative process from the initiation of data collection to the study’s end. CL kept a reflexive journal, recording entries after each focus group and interview to check for potential biases as a clinician/researcher and to identify recurring thoughts and ideas from discussions. Transcripts were manually analysed independently by two researchers (CL and KM, an experienced qualitative researcher) using a generic inductive thematic approach as described by Patton [19] and Harding [21]. After immersion into the data by iteratively reading the transcripts, the researchers identified primary patterns and coded the data in a constant comparative manner, with cross-referencing made to notes from CL’s reflexive journal. The codes were then categorised into themes [19, 21, 22]. Consensus concerning the emerging themes was then reached between the two researchers (CL and KM) with any conflicts resolved through discussion with a third researcher (DI).

## Results

A total of 65 primary and tertiary health professionals participated in 10 focus groups and 8 semi-structured interviews. The composition of the focus groups and demographic characteristics of participants is provided in Tables 1 and 2. No single health professional dominated focus groups discussions, with all participants contributing, although one allied health participant in one group was less contributory.

**Table 1:** Characteristics of general practitioner focus groups

Health region focus group (FG)	GP FG 1	GP FG 2	GP FG 3	GP FG 4	All
Mean age range (intervals of 10 years)	50–60	60–70	50–60	50–60	50–60
Male (Female)	6 (0)	6 (0)	4 (0)	4 (2)	20 (2)
Solo practice (n)	0	3	2	0	5
Number of participants	6	6	4	6	22

**Table 2:** Characteristics of tertiary health professional focus groups

Focus group	Hospital	Health professionals involved (n)	Gender (n) of involved health professionals	Male: Female ratio for the entire group	Total (n)
THP FG 1	Hospital A	Endocrinologists (2)	Female (2)	1:7	8
		Nephrologists (2)	Male (1); Female (1)		
		Diabetes nurse practitioner (1)	Female (1)		
		Diabetes nurse educator (1)	Female (1)		
		Renal nurse practitioners (2)	Female (2)		
THP FG 2	Hospital A	Social Workers (3)	Male (1), Female (2)	1:2	3
THP FG 3	Hospital B	Endocrinologist (1),	Female (1)	3:3	6
		Nephrologists (2),	Male (2)		
		Diabetes nurse educator (1),	Female (1)		
		Renal nurse (1),	Female (1)		
		Dietician (1),	Female (1)		
		Social worker (1)	Male (1)		
THP FG 4	Hospital C	Endocrinologist (1)	Female (1)	1:5	6
		Endocrine advanced trainee (1)	Female (1)		
		Diabetes nurse practitioner (1)	Female (1)		
		Renal nurse practitioner (1)	Female (1)		
		Dietician (1)	Female (1)		
		Nephrologist (1)	Male (1)		
THP FG 5	Hospital D	Nephrologist (1)	Male (1)	1:5	6
		Renal advanced trainee (1)	Female (1)		
		Renal nurses (2)	Female (2)		
		Dietician (1)	Female (1)		
		Diabetes nurse practitioner (1)	Female (1)		
THP FG 6	Hospital D	Diabetes nurse educators (2),	Female (2)	2:4	5
		Endocrine advanced trainees (4).	Male (2), Female (2)		
Total			N/A	9:26	35

**Fig. 1:**
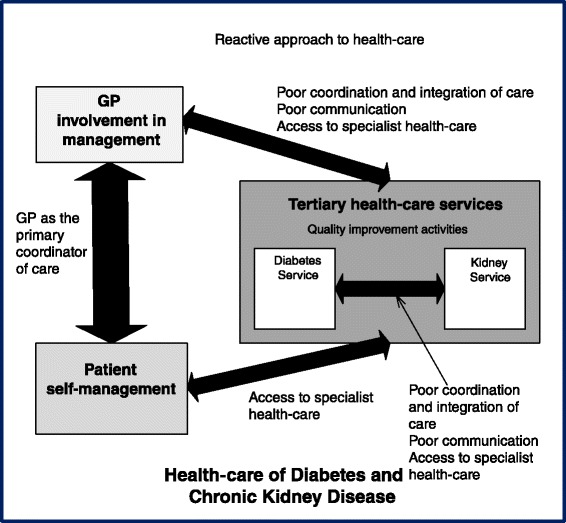
Thematic Schema illustrating health professionals’ views on factors influencing the health-care of co-morbid diabetes and chronic kidney disease

Four key factors were considered by all participants (including tertiary health professionals, heads of units and GPs) to influence health-care of co-morbid diabetes and CKD including self-management, access, coordination and integration of care, and a reactive approach to health-care (Table 3). An additional key factor (quality improvement activities) was identified through interviews with physicians who were the heads of unit. Management of diabetes and CKD was perceived to be an interaction between patient self- management and GP or primary care management, with ancillary specialist hospital or tertiary health-care management. A thematic schema illustrating the interplay between each factor is shown in Fig. 1. Further variation in themes according to health professional grouping (e.g. physician, nurse and allied health staff) were not apparent.

**Table 3:** Key factors seen to influence health-care of co-morbid diabetes and CKD

	Participant subgroup	
	All participants (including tertiary health professionals, heads of units and GPs)	Heads of units
Key factors	Self-management	Quality improvement activities
	Access to specialist health-care	
	Coordination and integration of care i) GP as the primary coordinator of care ii) Poor coordination of care and iii) Poor communication between health-care providers	
	Reactive approach to health

### Self-management

All health professionals thought that self-management by patients of co-morbid diabetes and CKD was essential for optimal health-care. Both GPs and tertiary health professionals felt that some patients relied on health professionals to manage their co-morbid diabetes and CKD instead of taking ownership and responsibility for their condition and self-managing.

*“Sometimes (…) patients remove themselves from any level of responsibility for their care.” – THP FG 5*

Several factors were purported to influence patients’ ability to self-manage. These included their understanding of illness, education, social factors (including culture and language), financial situation, psychological factors and co-morbidities, and their physical/mental aptitude. Health professionals emphasised the importance of patients’ understanding of diabetes and co-morbid CKD, its treatment, and its complications in promoting self-management, such as self-monitoring of blood glucose, taking medications and making dietary and lifestyle modifications.

*“I think that a lot of patients just simply do not realise the enormity of their condition. For some of them, they think its just a little bit of sugar.(…) They don’t associate diabetes with blindness (…) with CKD. They don’t associate diabetes with peripheral arterial disease, amputations etc. They just don’t appreaciate – and maybe some us might not either. They just don’t appreciate how dire their disease can be if they don’t look after themselves as well as they should”.* – GP FG 2

Most GPs and tertiary health professionals thought that patient self-management could be improved through education to improve patient understanding of co-morbid diabetes and CKD and better communication of medical information. Tertiary health professionals suggested that education and communication could be more patient-centred for example simpler, non-condescending, in the native language, culturally relevant, address incorrect beliefs of disease, and involve motivational interviewing/behavioural change/health coaching. They recommended that education be given early in the disease and also provided to family members. Additionally, GPs suggested that self-management could be fostered by giving patients a copy of their chronic care plan with treatment targets, current results and medical/allied health appointments.

### Access to tertiary health-care

Access to specialist tertiary health-care was considered to be challenging for both patients and other health professionals wanting advice.

For patients, tertiary health professionals thought that access to health-care for co-morbid diabetes and CKD could be difficult due to tertiary health-care being centralised in large hospitals, the lack of available appointments, long waiting times in clinic, the cost of travelling and medications, the time involved in treatment and the timing of treatment.

*“(…) for many people, coming into the hospital clinic, it is overwhelming in itself. It’s difficult to park, it’s difficult to get to, it’s busy.” -* THP FG 1

Similarly, all GPs complained about the long waiting times for initial patient reviews by specialist tertiary health-care diabetes or renal clinics or allied health services (frequently months) and the long waiting room times for patients.

*“They’re going to get a letter and they’re going to say in two weeks’ time you have an appointment in our clinic, but make sure you’re there half an hour early, but dedicate the whole afternoon because you may not leave until 5 pm. That’s what they get, so they take a cut lunch and thermos”.* – GP FG 3

Both tertiary health professionals and GPs agreed that access to tertiary health-care advice for health professionals was problematic. They reported that referral processes were difficult, complicated and tedious. GPs were often unsure if their referrals had been received and what the waiting times before review would be. Others indicated wanting more information about the specialist tertiary health-care services offered by hospitals and being unsure about referral guidelines and pathways to the diabetes or renal clinics.

*“There’s no actual easy centralised process where we can make referrals”.* – THP FG 4

GPs reported difficulty clarifying management decisions made during tertiary health-care clinics through phone calls and frustration that calls were either not returned or returned after a long delay.

*“That sort of bureaucratic interface which often doesn’t work, and it’s not often the clinics, it’s the larger hospital administrative interface where just trying to get through to talk to somebody rather than an answering machine and getting someone to come back to you and trying to convey a sense of urgency”. – GP* FG 4

Conversely, most heads of units thought that their services provided access to good quality, individualised, patient-centred care at a low cost (since this was covered by the government).

All GPs and tertiary health professionals agreed that there was insufficient access to tertiary health-care level allied health (especially dietetics, psychology, social work, podiatry, and in some cases even diabetes education) and ophthalmology services. Most GPs found accessing allied health and diabetes nurse educator services from tertiary health-care services frustrating. They felt that tertiary health-care services were generally unwilling to offer allied health services to patients referred by GPs.

Both GPs and tertiary health professionals felt that access to specialist tertiary health-care services could be improved by a health professional triaging referrals and acting as a point of phone contact for referring GPs and other tertiary health professionals. This would facilitate phone consultations about potential referrals, and clarification of treatment decisions made in specialist tertiary health-care clinics. They also suggested that the referral process could be simplified with greater clarity surrounding referral criteria, and improved communication concerning reception of the referral and waiting-times prior to clinic review.

Decentralising tertiary health-care services to the community could improve access. Some GP and tertiary health professionals suggested that outreach clinics could be located in community centres serviced by GPs with an interest in managing diabetes and CKD, with specialist physicians having a supportive and educational role. This would not only foster better relationships and communication between tertiary health professionals and GPs, but upskill GPs in managing diabetes and CKD.

### Coordination and integration of care

Three sub-themes are covered by the larger theme of coordination and integration of care. These are 1) the GP as the coordinator of care 2) poor coordination of care across tertiary and primary health-care 3) poor communication across tertiary and primary health-care

#### The GP as the coordinator of care

A major sub-theme emerging was the role of the GP as the coordinator of care for patients with co-morbid diabetes and CKD, with specialist services playing an ancillary role. While both GPs and tertiary health-care professionals generally agreed with this in principle, there were several barriers to this that were expressed.

First, there was distrust between tertiary health professionals and GPs. Many tertiary health professionals thought that GPs lacked expertise and required more education in managing diabetes and CKD to prevent inappropriate management. They thought that GPs either over-referred or didn’t prioritise managing diabetes and preventing complications, resulting in late referrals to tertiary health-care. This resulted in many tertiary health-care services coordinating the care of patients instead of relying on the GP.

*“I think because diabetes is so complex in terms of it being managed by poly pharmacy that that’s very difficult for the – yeah, I think that’s very difficult for GPs to do really well. And it’s important that people are medically managed (…) very well [so] we [can] help achieve good control… also when you put kidney disease into the background then a lot of the diabetes medications need to be reviewed. I think that’s problematic. And the difficulty too with the GP starting someone on insulin is huge, because they don’t have anyone out there to help them do it” –* THP FG 5

On the other hand, GPs noted that tertiary health services would often takeover the diabetes and CKD care of their patients. They felt that tertiary services were dismissive of their roles as the primary coordinator of care and unfairly under-estimated the expertise and willingness of GPs to manage diabetes and CKD due to previous poor experience with other GPs.

*“And you do get judged by your lowest common denominator (…) you only need one or two bad stories and then that sets a reputation within the system that ‘We don’t trust GPs’ or ‘GPs don’t do this well’”.* – GP FG 4

Many thought that some tertiary services performed primary care roles such as coordinating patient care or managing simple, early diabetes and CKD.

Second, tertiary health professionals reported difficulty when patients did not have a regular GP or had very little contact with their GP. One possible reason cited by health professionals was that some patients had so many medical appointments (including dialysis) due to their multi-morbidity that they could not maintain contact with their GP.

In order to facilitate GPs being the primary coordinator of care for co-morbid diabetes and CKD, both GPs and tertiary health professionals recommended greater role clarification and collaboration and education and upskilling of GPs in the management of and indications for specialist referral of co-morbid diabetes and CKD. GPs suggested that this could be achieved through obligatory training on diabetes and CKD supported by financial incentives; better dissemination of standardised diabetes and CKD health-care guidelines; secondment to specialist diabetes and/or CKD clinics; and attendance at Diabetes and Renal Unit meetings. GPs also suggested that they should be encouraged to manage chronic diseases more (such as diabetes and CKD) through financial incentives.

#### Poor coordination and integration of health-care

The second sub-theme raised by both GPs and tertiary health professionals, was poor coordination of care across primary and tertiary health-care. Tertiary health professionals described health-care at the tertiary level as siloed and fragmented into different specialties. Both GPs and tertiary health professionals reported an insufficient level of integration of care between tertiary and primary levels of care. They associated poor coordination of care with a lack of coordination of appointments for patients, and a lack of clarification of roles resulting in duplication of tests and treatment, conditions being left untreated (due to the erroneous assumption that another health professional would manage the condition), conflicting messages given to patients, and frequent multiple changes in treatment.

*“Sometimes patients get completely confused because you’ve made one change, someone’s made the other change and sometimes you don’t know who’s making the change (…) the communication about who is doing what management is not clear.” – THP FG 1*

*“Often I’ll send them in with all their blood tests and they’ll immediately do another set at the hospital”.* – GP FG1

Most GPs and tertiary health professionals suggested that coordination and integration of health-care for co-morbid diabetes and CKD could be improved through a multidisciplinary diabetes and renal specialist service which would combine several specialty appointments into one visit, improving integration of care and decreasing the number of clinic appointments for patients. Tertiary health professionals spoke about this in depth, suggesting that the clinic could offer structured care and be staffed by endocrinologists, nephrologists, social workers, dieticians, diabetes and renal nurses and psychologists, with other staff such as podiatrists, ophthalmologists, pharmacists, and vascular surgeons being involved on an on-call basis. Both GPs and tertiary health professionals saw 2 roles for such a clinic – 1) chronic disease management support for those with diabetes and CKD and a complex medical/social history such as those on dialysis or with a kidney transplant 2) crisis support and a consultative service for patients which GPs had trouble managing. However, some GPs cited logistical difficulties in implementation of such a clinic or the clinic having too narrow a focus.

To improve coordination of care and clarification of roles, especially between primary and tertiary health-care, many GPs and tertiary health professionals proposed a structured shared care pathway or care plan outlining management goals, with agreed predefined roles for the GP and the specialist physicians and schedule of appointments with involved health professionals, individualised to the patient’s severity of co-morbid diabetes and CKD. They also suggested multidisciplinary case conferencing for difficult patients.

#### Poor communication between health-care providers

All participants highlighted the importance of effective communication of medical information between health providers (including specialist physicians, GPs and providers of pathology or radiology services). However, they perceived communication to be often poor between and within all levels of care, contributing to poor coordination of care.

Tertiary health professionals and GPs complained about poor communication of referrals, management decisions and investigations between each other. While tertiary health professionals complained that referral letters from GPs often containing inadequate information. Most GPs and some tertiary health professionals also suggested that communication of management decisions back to GPs was poor. GPs reported that this was especially a problem with renal and dialysis services and recounted difficulties in patient care due to delayed (one GP mentioned up to 3 weeks) or no correspondence being sent by tertiary health-care clinics.

*“There’s not much happening from the GP’s end to us, in particular whatever correspondence we see in the clinics (…). The only information we get (…) is they just make sure they get a referral to come and see the endocrinologist (…) and then you pretty much gather the information from the patient.” - THP* FG 6

*“Unfortunately, there’s a pretty big disconnect between primary practice and tertiary. There still is. There probably always will be because – there are some units which are very good at communicating with me and try quite earnestly to keep in contact, but other ones who don’t”.* – GP FG 2

Tertiary health professionals identified problems with the main communication pathways - letters and medical records. Letters were reportedly sent out late. There were also identified problems with the medical records system (scanned medical records on an information technology platform) which were often not integrated between departments (with investigation results in different databases), incomplete (with delays in scanning clinic notes into the system, and the absence of a current medication lists), difficult to read (due to handwritten notes being scanned) and difficult to navigate.

Both GPs and tertiary health-care professionals suggested that an integrated electronic medical record that was shared, accessed and updated real-time by all health professionals in tertiary and primary care could improve communication between health professionals. The medical record would ideally contain an updated medical summary and medication list. Further, a policy of mandatory and timely communication of management decisions to the coordinating GP and all involved health providers could be implemented to improve communication

### A reactive approach to health

A reactive approach to health-care of co-morbid diabetes and CKD, whereby health-care was focused on fixing problems rather than preventing disease and complications, was identified to be a problem by GPs and tertiary health professionals at both primary and tertiary health-care levels. Related issues of therapeutic inertia, failure to reach clinical performance targets and late referrals to nephrology or endocrinology were identified as barriers to care.

Tertiary health professionals articulated that a reactive approach within tertiary health-care was fostered by limited resources including limited time, high patient-doctor ratios and lack of physical space to house more doctors. This led them to deal with and manage the most pressing issues, especially for those patients with multi-morbidity, instead of taking a thorough preventive approach to health-care.

*“For the patients that can get here, they are complex patients, they need a lot of time, (…) they need a lot of motivation and encouragement and positive feedback and clinics are busy and there’s not time to do all of that really, and we tend to focus on (…) what needs to be fixed.” –*THP FG 3

Similarly, GPs reported treatment and management gaps with poor glycaemic control of diabetes and inadequate screening for diabetes complications such as CKD in primary care.

Both GPs and tertiary health professionals expressed the importance of preventing diabetes, and preventing the onset of CKD in diabetes.

*“Until we focus on prevention and making people leaner, we’re not going to succeed”-* GP FG 4

They recommended the introduction of public health preventive measures to raise awareness of and help prevent diabetes and CKD and CKD in diabetes. Some GPs even suggested celebrity sponsors to help raise awareness.

### Quality improvement activities

Almost all unit heads expressed a need for greater engagement in quality improvement activities in their services. They perceived that a lack of evaluation and quality improvement activities accompanied by a lack of performance feedback led to poorly managed co-morbid diabetes and CKD.

*“And anything that we do – if we make changes we need to be able to audit that. We need to be able to follow it, we need to be able to make improvements on that basis”.* – FG 5

Some suggested quality improvement activities, including a database to allow regular audits of health service performance and clientele profiles, and ultimately identify service issues which could then be targeted and improved.

## Discussion

Co-morbid diabetes and CKD, with its associated health burden and health-care costs [5, 23], is an exemplar of the challenge facing health-systems with multi-morbidity, where the priority of health-care and research has been focussed on a single disease [2, 13]. In this wide reaching and novel qualitative study of both GPs and tertiary health professionals across 4 large health services, we report factors identified to influence the health-care of co-morbid diabetes and CKD. We found that both GPs and tertiary health professionals perceived that health-care of co-morbid diabetes and CKD was an interaction between a patient’s self- management and primary health-care management with ancillary tertiary health-care support. Patient self-management, access, coordination and integration of care, a reactive approach to health and quality improvement activities were important factors for optimal health-care of co-morbid diabetes and CKD. Differences in opinion between GPs and tertiary health professionals centred around problems underlying poor coordination and integration of care, with a mistrust between these groups hindering GPs acting as the primary coordinator of care and each group blaming the other for poor communication.

Our qualitative study adds to existing data by exploring the views of both GPs and tertiary health professionals on health-care for co-morbid diabetes and CKD. Previous qualitative studies involving health professionals have examined the health-care of diabetes [24, 25] or CKD [26] as distinct disease entities or chronic disease in general [27]. While some of the reported themes are similar to our findings (such as the need for better integration and coordination of care [24, 27]), the development and improvement of health systems to deal with multi-morbidity must be tailored to formative research identifying the unique challenges for the relevant population [28], in this case patients with co-morbid diabetes and CKD.

Improving patients’ self-management is important in any chronic disease, and arguably even more important in the setting of multi-morbidity. A pre and post design study evaluating an education program designed to improve self-efficacy and self-management skills of patients with diabetic nephropathy reported improvement in glycaemic control and maintenance of renal function [29]. The emphasis on self-management from our qualitative study reinforces the importance of self-management and this should be a vital component of any health-care improvement initiative.

Participants in our study highlighted the issue of difficult access to specialist services. Both GPs and tertiary health professionals proposed that access could be improved by decentralising specialist care to the community and having outreach specialist clinics specialists in GP practices (which had the additional purported benefit of improving GP knowledge). While there is data supporting decentralisation of tertiary health-care services for other chronic diseases and settings [30–32] the benefit of such an approach for co-morbid diabetes and CKD will need to be confirmed.

Coordination and integration of care was an important factor highlighted by both primary and tertiary health-care professionals. In particular both groups agreed in principle that the GP should be the coordinator of care. However, there appeared to be a mistrust between GPs and tertiary health professionals hindering this from occurring with GPs reporting tertiary health professionals taking over primary care issues and tertiary health professionals doubting the ability of GPs to adequately manage patients. This mistrust will have to be overcome with better communication and greater role clarification if GPs are to function as the primary coordinator of care. The importance of GPs as the coordinator of care in general has been recognised by the patient-centred medical home (PMCH) model of care, which emphasises improved coordination of care in health service delivery around primary care [33]. Implementation of the PCMH in other settings has improved patient satisfaction, clinical quality and decreased health-care utilisation (hospitalisation rates [34] and emergency visits [35]), and care costs [36].

The importance of coordination and integration between primary and tertiary care in the form of shared care has previously been reported to improve glycaemic control and recommended monitoring [37]. In our study, many participants suggested that a structured diabetes-renal shared care plan or pathway clarifying clinical care roles and goals, could facilitate this, and improve communication. Similar tools have been shown to be beneficial in CKD care [38], but have not been trialled in co-morbid diabetes and CKD.

Improved coordination of care within specialist services via a multidisciplinary combined diabetes-renal service with structured care was suggested by participants in our study. Quality audit and observational studies of a combined diabetes and renal service in the United Kingdom have reported improvements in the rate of renal function decline and in treatment target attainment including HbA1c [10, 15]. Similarly, a randomised controlled trial of structured care in Hong Kong for diabetes and CKD reported reduced death and development of ESKD [16]. Our study supports these findings but suggests that proper integration and collaboration of such a service with primary health-care is paramount.

Our study’s qualitative approach enabled a detailed exploration of perceptions and ideas unachievable using a quantitative approach. The methodology and results are strengthened through the triangulation of the focus groups with semi-structured interviews, thematic analysis by two investigators and the use of reflexive journaling (ensuring credibility of the findings). Additionally, the use of maximal variation sampling of a relatively large number of information rich cases across a wide strata of health professional roles, in different geographical locations, increases transferability of findings. Conversely, while many of the issues raised are universal across health systems, the inclusion of Australian health professionals, may limit transferability of findings to other settings. A relative lack of female GP participants and excess of female tertiary health professional participants was noted. This could be a reflection of the medical workforce in Australia with 57 % of GPs being male and a third of female GPs working part-time [39] as well as a higher percentage of endocrinologists and nurses being female [39, 40]. We also acknowledge the inherent weakness of qualitative research including the potential for researcher and participant bias in both collection and analysis of the data, given that the researcher (CL) who moderated all focus groups, conducted all interviews, and analysed the data, was also an Endocrinologist. This was minimised by CL keeping a reflexive journal, and the thematic analysis being performed separately by two researchers (CL and KM, with KM being a non-clinical researcher) before consensus was reached concerning the emerging themes. In addition, an advantage of CL facilitating all focus groups and semi-structured interviews was that it provided consistency in data collection and enhanced interpretation of the data as interpretation of actions, body language and focus group participant interactions was possible.

## Conclusion

In conclusion, health professionals from both primary and tertiary health-care emphasise the importance of patient self-management and the central role of the GP in coordinating care. Key barriers to optimal health-care identified included poor coordination of care and communication, access to specialist health-care, and a reactive approach to health. Quality improvement activities were also seen to be important. Supporting primary health-care with an accessible, multidisciplinary combined diabetes-renal health service underpinned by strong communication pathways, a preventive approach and quality improvement activities, may improve health-care and outcomes for patients with co-morbid diabetes and CKD. Results from this study will be used to inform the co-design of a new patient-centred model of care for co-morbid diabetes and CKD which will then be implemented and evaluated.

### Ethics and consent to participate

This study was approved by all local hospital and university Human Research Ethics Committees (Monash Health Human Research Ethics Committee, Alfred Health Research Ethics Committee, Monash University Human Research Ethics Committee, Northern Sydney Local Health District Human Research Ethics Committee, Sydney Local Health District Human Research Ethics Committee and the University of Sydney Human Research Ethics Committee). All participants were involved voluntarily and gave written consent for participation in and audio-recording of focus groups or semi-structured interviews.

### Consent to publish

Not applicable

### Availability of supporting data

Transcripts for this study can be obtained by contacting Clement Lo at clement.lo@monash.edu.

## Additional file

Additional file 1: Table S1. Initial Questions inventory for focus groups and semi-structured interviews (DOCX 12 kb)

## Abbreviations

CKD: chronic kidney disease
FG: focus group
GP: general practitioner
SSI: semi-structured interview
THP: tertiary health professional

## References

[1] Global, regional, and national incidence, prevalence, and years lived with disability for 301 acute and chronic diseases and injuries in 188 countries, 1990–2013: a systematic analysis for the Global Burden of Disease Study 2013. Lancet. 2015. doi:10.1016/s0140-6736(15)60692-4 10.1016/S0140-6736(15)60692-4 PMC4561509 26063472

[2] Atun R (2015). Transitioning health systems for multimorbidity. Lancet.

[3] International Diabetes Federation 2013. IDF Diabetes Atlas. 6th Edition. Belgium: International Diabetes Federation, 2013.

[4] Tuttle KR, Bakris GL, Bilous RW, Chiang JL, de Boer IH, Goldstein-Fuchs J (2014). Diabetic kidney disease: a report from an ADA Consensus Conference. Diabetes Care.

[5] Foley RN, Murray AM, Li S, Herzog CA, McBean AM, Eggers PW (2005). Chronic kidney disease and the risk for cardiovascular disease, renal replacement, and death in the United States Medicare population, 1998 to 1999. J Am Soc Nephrol JASN.

[6] 2015 US Renal Data System Annual Data Report Volume 1: Chronic Kidney Disease in the United States. Ann Arbor, Michigan: USRDS Coordinating Center2015.

[7] Meyers JL, Candrilli SD, Kovacs B (2011). Type 2 diabetes mellitus and renal impairment in a large outpatient electronic medical records database: rates of diagnosis and antihyperglycemic medication dose adjustment. Postgrad Med.

[8] Allen AS, Forman JP, Orav EJ, Bates DW, Denker BM, Sequist TD (2011). Primary care management of chronic kidney disease. J Gen Intern Med.

[9] Hassan Y, Al-Ramahi RJ, Aziz NA, Ghazali R (2010). Adverse drug events in hospitalized patients with chronic kidney disease. Int J Clin Pharmacol Ther.

[10] Thabit H, Besharatian B, Conlon PJ, Smith D (2012). Complications and characteristics of patients referred to a joint diabetes renal clinic in Ireland. Ir J Med Sci.

[11] Lenz O, Mekala DP, Patel DV, Fornoni A, Metz D, Roth D (2005). Barriers to successful care for chronic kidney disease. BMC Nephrol.

[12] Kausz AT, Guo H, Pereira BJ, Collins AJ, Gilbertson DT (2005). General medical care among patients with chronic kidney disease: opportunities for improving outcomes. J Am Soc Nephrol.

[13] Barnett K, Mercer SW, Norbury M, Watt G, Wyke S, Guthrie B (2012). Epidemiology of multimorbidity and implications for health care, research, and medical education: a cross-sectional study. Lancet.

[14] Lo C, Ilic D, Teede H, Cass A, Fulcher G, Gallagher M et al. The Perspectives of Patients on Health-Care for Co-Morbid Diabetes and Chronic Kidney Disease: A Qualitative Study. (1932–6203 (Electronic)). doi:D - NLM: PMC4701448 EDAT- 2016/01/06 06:00 MHDA- 2016/01/06 06:00 CRDT- 2016/01/06 06:00 PHST- 2016 [ecollection] PHST- 2015/09/17 [received] PHST- 2015/12/18 [accepted] PHST- 2016/01/05 [epublish] AID - 10.1371/journal.pone.0146615 [doi] AID - PONE-D-15-40881 [pii] PST - epublish.

[15] Jayapaul MK, Messersmith R, Bennett-Jones DN, Mead PA, Large DM (2006). The joint diabetic-renal clinic in clinical practice: 10 years of data from a District General Hospital. QJM.

[16] Chan JC, So WY, Yeung CY, Ko GT, Lau IT, Tsang MW (2009). Effects of structured versus usual care on renal endpoint in type 2 diabetes: the SURE study: a randomized multicenter translational study. Diabetes Care.

[17] Cherryholmes CH. Educational Researcher. 1992;August - September:13–7.

[18] Creswell J (2014). Research Design: Qualitative, Quantitative and Mixed Methods Approaches.

[19] Patton MQ (2002). Qualitative Research and Evaluation Methods.

[20] Krueger RA, Casey MA. Focus Groups. A practical guide for applied research. 4th Edition. Thousand Oaks, California: Sage; 2009.

[21] Harding J (2013). Qualitative Data Analysis from Start to Finish.

[22] Miles MB, Huberman AM. Qualitative Data Analysis - An expanded sourcebook. Thousand Oaks, California: Sage, 1994

[23] Gordois A, Scuffham P, Shearer A, Oglesby A (2004). The health care costs of diabetic nephropathy in the United States and the United Kingdom. J Diabetes Complications.

[24] Peytremann-Bridevaux I, Lauvergeon S, Mettler D, Burnand B (2012). Diabetes care: Opinions, needs and proposed solutions of Swiss patients and healthcare professionals: a qualitative study. Diabetes Res Clin Pract.

[25] Lauvergeon S, Mettler D, Burnand B, Peytremann-Bridevaux I (2012). Convergences and divergences of diabetic patients’ and healthcare professionals’ opinions of care: a qualitative study. Health Expect Int J Public Participation Health Care Health Policy.

[26] Blakeman T, Protheroe J, Chew-Graham C, Rogers A, Kennedy A (2012). Understanding the management of early-stage chronic kidney disease in primary care: a qualitative study. Bri J General Practice J Royal College General Practitioners.

[27] Van Durme T, Macq J, Anthierens S, Symons L, Schmitz O, Paulus D (2014). Stakeholders’ perception on the organization of chronic care: a SWOT analysis to draft avenues for health care reforms. BMC Health Serv Res.

[28] Kochevar LK, Yano EM (2006). Understanding health care organization needs and context. Beyond performance gaps. J Gen Intern Med.

[29] Kazawa K, Moriyama M (2013). Effects of a self-management skills-acquisition program on pre-dialysis patients with diabetic nephropathy. Nephrol Nurs J J Am Nephrol Nurses’ Assoc.

[30] Sibbald B, Pickard S, McLeod H, Reeves D, Mead N, Gemmell I (2008). Moving specialist care into the community: an initial evaluation. J Health Serv Res Policy.

[31] Bowling A, Bond M (2001). A national evaluation of specialists’ clinics in primary care settings. Bri J General Practice J Royal College General Practitioners.

[32] Gruen RL, Weeramanthri TS, Knight SE, Bailie RS (2004). Specialist outreach clinics in primary care and rural hospital settings. Cochrane Database Syst Rev.

[33] The Medical Home (2014). What do we know, what do we need to know?.

[34] Nelson KM, Helfrich C, Sun H, Hebert PL, Liu CF, Dolan E (2014). Implementation of the patient-centered medical home in the veterans health administration: associations with patient satisfaction, quality of care, staff burnout, and hospital and emergency department use. JAMA Int Med.

[35] David G, Gunnarsson C, Saynisch PA, Chawla R, Nigam S (2014). Do Patient-Centered Medical Homes Reduce Emergency Department Visits?. Health Serv Res.

[36] van Hasselt M, McCall N, Keyes V, Wensky SG, Smith KW (2014). Total Cost of Care Lower among Medicare Fee-for-Service Beneficiaries Receiving Care from Patient-Centered Medical Homes. Health Serv Res.

[37] Ciardullo AV, Daghio MM, Brunetti M, Bevini M, Daya G, Feltri G (2004). Audit of a shared-care program for persons with diabetes: baseline and 3 annual follow-ups. Acta Diabetol.

[38] Haley WE, Beckrich AL, Sayre J, McNeil R, Fumo P, Rao VM (2015). Improving care coordination between nephrology and primary care: a quality improvement initiative using the renal physicians association toolkit. Am J Kidney Dis.

[39] ABS. Australian Social Trends, April 2013 - Doctors and Nurses http://www.abs.gov.au/AUSSTATS/abs@.nsf/Lookup/4102.0Main+Features20April+2013#p8 (accessed 22 January 2016). Australian Bureau of Statistics. 2013. Accessed 22 January 2016

[40] ABS. Australian Social Trends, April 2013 - Increase in number of young female doctors http://www.abs.gov.au/ausstats/abs@.nsf/products/BF9BEBC9B854646ACA257BB200142357?OpenDocument (accessed 22 January 2016). Australian Bureau of Statistics. 2013. Accessed 22 January 2016

