# Supplementary material for: Primary and tertiary health professionals’ views on the health-care of patients with co-morbid diabetes and chronic kidney disease – a qualitative study
Source: BMC Nephrol. 2016 May 18;17:50. doi: 10.1186/s12882-016-0262-2 (PMC4870736; doi:10.1186/s12882-016-0262-2)
Supplement: Additional file 1: Table S1. — Initial Questions inventory for focus groups and semi-structured interviews (DOCX 12 kb) [file 12882_2016_262_MOESM1_ESM.docx]

**Supplementary Materials**

**Table S1: Initial Questions inventory for focus groups and semi-structured interviews**

| 1 | From your previous experiences, what factors (individual, environmental, family, social, health system) do you think contribute to patients with diabetes and chronic kidney disease having good health outcomes? |
| --- | --- |
| 2 | From your previous experiences, what factors (individual, environmental, family, social, health system) do you think contribute to patients with diabetes and chronic kidney disease having poorly managed DM and CKD |
| 3 | In your opinion, what do you think are the strengths of the current health services for patients with DM and CKD? |
| 4 | What do you find particularly effective about the processes and the way that current health services for patients with DM and CKD are run? |
| 5 | What are weaknesses of the current services for patients with DM and CKD? |
| 6 | What do you find particularly frustrating about current services for patients with DM and CKD? |
| 7 | How accessible do you think current health services are to patients? |
| 8 | Taking into account realistic funding opportunities, in your opinion, what realistically would an ideal health-care model look like for patients with DM and CKD?  What would be the key components of such a model?  How can the health service move from the current service provision to a different model? |
| 9 | We want you to help us evaluate these services to help improve the service and the difference it makes to patients. Is there anything that we have missed or that I should’ve asked you that I didn’t think to ask? |
